# Food Agency and Intentions to Improve Cooking Skills Among Korean Young Adults: Demographic Differences and Motivators

**DOI:** 10.3390/nu18040656

**Published:** 2026-02-17

**Authors:** So-Young Kim, Ji Yu Choi, Min Hyun Maeng

**Affiliations:** 1 Department of Food Science and Nutrition, Soonchunhyang University, Asan 31538, Chungcheongnam-do, Republic of Korea; 2 Department of Food and Nutrition, Pai Chai University, Daejeon 35345, Chungcheongnam-do, Republic of Korea; 3 Department of Consumer Behavior and Family Economics, University of Wisconsin, Madison, WI 53706, USA; mmaeng@wisc.edu

**Keywords:** food skills, cooking practices, cooking competency, early adulthood

## Abstract

**Background/Objectives**: This study aimed to examine levels of food agency and intentions to improve cooking skills among Korean young adults in their 20s, and to identify demographic differences and underlying motivators. **Subjects/Methods**: An online survey was administered from February 18 to 25, 2021, targeting Korean adults aged 20–29 years (*n* = 1102; 579 men, 523 women). Food agency was assessed using the Cooking and Food Provisioning Action Scale (CAFPAS), comprising three subscales: self-efficacy (13 items), attitude (10 items), and structure (5 items). Participants also reported their intentions to improve cooking skills and the motivators behind those intentions. Reliability of multi-item measures was confirmed using Cronbach’s alpha. Descriptive statistics were calculated for all study variables. Demographic differences in food agency and intentions were analyzed using independent *t*-tests and one-way ANOVA. Open-ended responses were analyzed using qualitative content analysis to explore key motivators. Statistical analyses were performed using SPSS Statistics 28.0 (α = 0.05). **Results**: Food agency scores were significantly lower among university graduates, employed individuals, and those from lower-income households. Single-person households reported significantly higher self-efficacy and attitude scores, while structure scores were significantly lower in this group. Lower structure scores were also observed among women; university graduates or individuals with higher levels of education; employed respondents; and those belonging to the low-income group. Intentions to improve cooking skills were significantly higher among women and single-person households. Qualitative analysis identified media influence and the demands of independent living as primary motivational drivers. **Conclusions**: These findings highlight the need for targeted interventions to enhance structural capacity for food agency, particularly among women, employed individuals, those with higher education, and people in the low-income range. Efforts should focus on leveraging media influence and supporting individuals adapting to independent living to promote cooking skill development.

## 1. Introduction

Cooking skills are widely recognized as essential life skills that support dietary autonomy and overall well-being. Traditionally, these skills were passed down through family traditions or taught formally through school-based home economics curricula. However, in recent decades, growing concern has emerged over “culinary deskilling”—a societal trend in which individuals, particularly younger generations, are losing basic cooking competencies. This decline has been attributed to shifts in lifestyle patterns, increased availability of ready-to-eat meals, and limited opportunities for hands-on cooking education [1].

Young adults in their 20s are undergoing a transitional life phase marked by increased independence, such as leaving home, pursuing higher education, or entering the workforce. Dietary practices established during this stage tend to persist into later adulthood [2], underscoring the importance of understanding cooking-related practices during this life phase. Nevertheless, young adults are frequently identified as a group with unstable and suboptimal dietary patterns [3,4,5].

In South Korea, rapid modernization and dietary westernization have significantly altered traditional food practices among young adults. Despite these shifts, empirical evidence on the cooking-related practices of young adults remains limited. To address these limitations, the present study adopts the concept of food agency, which provides a more comprehensive framework for understanding cooking-related practices by integrating individual skills, attitudes, and structural conditions. Using the Cooking and Food Provisioning Action Scale (CAFPAS) developed by Lahne et al. [6], this study aims to (1) examine levels of food agency among Korean young adults in their 20s and (2) explore motivations underlying their intention to improve cooking skills. The findings are expected to inform evidence-based interventions and policies that promote cooking-related practices and cooking skills as a critical component of lifelong health and self-sufficiency.

## 2. Literature Review

### 2.1. Cooking Skills, Diet Quality, and Health Outcomes in Early Adulthood

Despite limitations in theoretical grounding, existing studies have consistently emphasized the multiple benefits of cooking and home meal preparation. These include fostering healthier food choices, reducing reliance on highly processed convenience foods [7], and ultimately contributing to improved dietary quality and long-term health outcomes [8,9,10]. Conversely, insufficient cooking skills have been associated with increased consumption of highly processed convenience foods [11], which may negatively affect diet quality and elevate the risk of diet-related health problems over time.

Early adulthood represents a critical life stage in this relationship. As young adults transition to independent living, they assume primary responsibility for food provisioning, often under conditions of time scarcity, financial constraint, and competing academic or occupational demands. During this period, cooking-related practices play an important role in supporting self-sufficiency and the ability to manage one’s own diet.

Empirical evidence indicates that cooking self-efficacy and engagement in home meal preparation are positively associated with healthier eating practices [10,12]. Seeley et al. [13] emphasized that cooking should not be viewed as an end in itself, but rather as a means to achieve and maintain healthy eating patterns. Mastery of cooking and related functional skills—such as food planning, shopping, and navigating food environments—can therefore facilitate dietary independence during early adulthood. Given that dietary patterns established during this life stage tend to persist into later adulthood, understanding cooking-related practices and the factors that shape them is essential for informing early preventive health interventions.

### 2.2. Food Agency as a Framework for Cooking-Related Practices

Although the term food agency has not yet been widely used in Korean literature, it provides a comprehensive framework for understanding cooking-related practices, particularly individuals’ ability to plan, procure, and prepare food in accordance with their values, knowledge, and situational constraints. In this study, food agency is adopted both conceptually and operationally to assess young adults’ cooking-related practices.

Food agency refers to an individual’s capacity to acquire and enact manual and cognitive skills within physical, economic, and social contexts during meal preparation, emphasizing empowerment in pursuing food-related goals rather than the mere acquisition of discrete cooking skills [14,15]. Lahne et al. [6] further conceptualized food agency as comprising three interrelated dimensions: self-efficacy, reflecting confidence in one’s ability to cook; attitude, capturing enjoyment of and positive orientations toward cooking; and structure, denoting the ability to manage contextual constraints such as time scarcity, financial limitations, and competing responsibilities. Empirical studies applying the Cooking and Food Provisioning Action Scale (CAFPAS) have demonstrated the utility of this framework for understanding everyday food practices across diverse populations [15,16].

Building on this conceptualization, intervention-oriented studies have examined how food agency responds to targeted programs in different demographic and contextual settings, frequently operationalizing the construct using CAFPAS. Evidence from these studies suggests that while certain dimensions of food agency are responsive to intervention, others remain constrained by broader structural conditions.

For example, in the United States, a meal-kit delivery intervention significantly increased food agency among college students [17]. The provision of meal kits, recipes, and complementary educational components led to statistically significant improvements in the self-efficacy and attitude subscales, whereas no significant changes were observed in the structure subscale. This pattern indicates that interventions may strengthen confidence and positive orientations toward cooking, yet are less effective in modifying structural barriers such as limited time and competing demands.

Findings from other contexts further underscore the role of structural constraints in shaping food agency. In Australia, fathers of children aged up to six years exhibited relatively high levels of food agency and shared food-related responsibilities, including meal planning, food shopping, and cooking; however, their involvement in these practices was shaped by time constraints associated with paid employment, as captured by the structure subscale [18].

### 2.3. Motivations Influencing Intention to Improve Cooking Skills

Prior research has identified multiple motivational factors influencing individuals’ engagement in cooking and their intention to improve cooking skills. These motivations encompass functional, psychological, and social dimensions, including self-fulfillment, enjoyment, health considerations, and cost-related concerns.

Focusing on young adults, Namin et al. [19] examined how self-fulfillment, hedonic, and social motivations shape college students’ decisions to cook at home versus dining out in metropolitan areas of the United States. Self-fulfillment motivation increased the perceived utility of cooking and was positively associated with home cooking. In contrast, hedonic motivation—linked to relaxation, pleasure, and recreation—was negatively associated with cooking at home, as students who derived high hedonic utility from cooking tended to restrict it to special occasions or perceived barriers such as inadequate kitchen facilities. Social motivation, defined as cooking as an expression of group identity or a means of social interaction, was not supported as a significant predictor of cooking behavior.

Additional studies conducted across broader demographic groups have identified other salient motivations for cooking, including cost savings [20], health and well-being [21], and enjoyment [22]. In particular, the preparation of meals from raw ingredients has been shown to be primarily driven by concerns for health and well-being [21]. Enjoyment of cooking has also been found to be more strongly associated with higher cooking skills among men than women in Switzerland [7].

Within this motivational landscape, media has emerged as an increasingly influential context for shaping cooking-related motivations and learning, particularly among younger populations. While mothers have traditionally served as the primary source of cooking skills, individuals now increasingly turn to digital platforms such as YouTube, TikTok, and Instagram for culinary knowledge and inspiration [21,23]. For example, Raber et al. [24] reported that low-income mothers in the United States rely on online resources for meal planning, cooking, and skill development, while Camargo et al. [25] found that young Brazilian adults engage with cooking-related content for both entertainment and learning purposes.

The appeal of digital media lies in its accessibility and flexibility, enabling users to learn at their own pace, explore diverse cuisines, and acquire techniques beyond traditional household practices. Such platforms may enhance culinary self-efficacy and stimulate intentions to cook [24,26]. However, media exposure also carries potential risks. In South Korea, frequent viewing of mukbang and cookbang content has been associated with unhealthy eating behaviors, including late-night eating and increased consumption of fast food and sugar-sweetened beverages [27]. Similarly, Cheng et al. [28] found that recipes high in fat and sugar generated greater engagement on Pinterest, incentivizing content creators to prioritize popularity over nutritional quality. These findings highlight the importance of critical engagement with online food content in shaping cooking-related motivations and behaviors.

## 3. Methods

### 3.1. Study Design and Participants

An online survey was conducted from 19th to 24th February 2021 among young Korean adults in their 20s and 1102 responses were collected. To enhance the validity of the questionnaire and check for central tendency in the responses, a pilot survey was conducted with 20 online survey panels from 8th to 9th February 2021 prior to the main survey.

Participant recruitment and data collection for both the pilot and main surveys were managed by Macromill Embrain, a leading online research firm in South Korea with a nationwide panel of over 1.5 million registered individuals. The company was responsible for website construction, participant recruitment, and survey administration. Participants were selected from the panel using a nonprobability sampling approach, specifically convenience sampling. To qualify for participation, individuals were required to (1) be aged between 20 and 29 years and (2) not have dietary restrictions related to medical conditions.

The final analytic sample consisted of 1102 respondents, which is considered adequate for population-based descriptive analyses and group comparisons across key demographic characteristics. This sample size provides sufficient statistical power to detect meaningful differences in food agency scores between subgroups using independent *t*-tests and one-way ANOVA. Table 1 presents the socio-demographic characteristics of the study participants.

This study was approved by the Institutional Review Board of Human Subjects Research and Ethics Committees of the Soonchunhyang University (Approval No. 1040875-202001-SB-002; Date: 17 February 2021). Written informed consent was obtained from all participants prior to the survey.

### 3.2. Survey Questionnaire

The data analyzed in this study were collected as part of a broader survey that has been used in a previous publication. In the earlier study, selected items related to cooking-related practices were analyzed to examine their associations with healthy eating habits. In contrast, the present manuscript focuses on examining levels of food agency across demographic subgroups among Korean young adults in their 20s and exploring the motivations underlying their intention to improve cooking skills. To address these distinct research objectives, survey items concerning (1) food agency, (2) intention to improve cooking skills and its underlying motivators, and (3) socio-demographic characteristics were analyzed.

In this study, food agency is adopted both conceptually and operationally to assess young adults’ cooking-related practices, Food agency was measured using the Cooking and Food Provisioning Action Scale (CAFPAS) developed by Lahne et al. [6]. The CAFPAS comprises the three sub-scales of self-efficacy (13 items), attitude (10 items) and structure (5 items) to capture the participants’ ability to procure and prepare food within the contexts of their social, physical and economic environment [6].

The CAFPAS was translated into Korean using a parallel back-translation procedure to verify that the original meaning was retained. The CAFPAS was translated into Korean by the authors and a professional English editor translated the text back into English. Each survey item of the back-translated CAFPAS was compared against that of the original CAFPAS. Finally, some revisions were made to convey the Korean context. All items were measured by a five-point Likert scale, anchoring from ‘strongly disagree’ to ‘strongly agree’.

Item scores within individual sub-scale are standardized, and then, the standardized scores for three sub-scales are summed to calculate an overall score for food agency (e.g., cooking-related practices). Some items were coded in reverse when necessary.

Next, participants were presented with a five-point scale question assessing their intention to improve their cooking skills. They were then asked a yes/no question to determine whether they had a specific motivator for this intention. Those who responded “yes” were subsequently given an open-ended question, allowing them to articulate their motivations in their own words and provide more detailed, nuanced explanations. In addition, socio-demographic characteristics including gender, education level, household income level and household composition were collected.

### 3.3. Statistical Analyses

The reliability of the multi-item measurements was assessed using Cronbach’s alpha coefficients. To evaluate whether any items should be deleted, we examined multiple criteria, including overall Cronbach’s alpha values (with a target threshold of >0.6), changes in alpha if an item was deleted, and corrected item–total correlations (targeting >0.3). In addition to these statistical indicators, the conceptual integrity of the latent constructs was carefully reviewed to ensure that the removal of any items would not compromise the core dimensions of food agency, with expert judgment consulted where necessary.

Based on this evaluation, three items were removed to improve internal consistency: one item from the attitude subscale (“I prefer to spend my time on more important things than food”) and two items from the structure subscale (“I wish that I had more time to plan meals” and “my family responsibilities prevent me from having time to prepare meals”). After item deletion, the revised food agency scale demonstrated good to excellent internal consistency, with Cronbach’s alpha coefficients exceeding 0.7 across subscales (Appendix A).

Descriptive statistical analysis was performed on all study variables. The differences in food agency and intention to improve cooking skills across respondents’ demographic characteristics were analyzed by independent sample *t*-test and one-way ANOVA. All statistical analyses were performed using SPSS Statistics version 28.0 at the significance level of 0.05.

The open-ended responses regarding the motivators for improving cooking skills were analyzed using inductive content analysis [29,30]. One author conducted the analysis, which involved open coding, category creation, and abstraction based on content-characteristic words [29]. The preliminary coding schema was then reviewed by the second author. Any discrepancies were resolved through discussion until consensus was reached. Throughout the analysis, coding decisions and category definitions were documented and iteratively refined, creating an audit trail that captured key analytical decisions and revisions. Simple frequency counts and proportional percentages were used to indicate the proportion of comments addressing each analytical category, based on the assumption that issues raised more frequently reflected aspects of motivation perceived as important by respondents [29]. Direct quotations are included to exemplify key findings emerging from the data.

## 4. Results

### 4.1. Differences in Food Agency According to Participants’ Socio-Demographic Characteristics

Table 2 presents the differences in food agency according to participants’ socio-demographic characteristics. Significant differences in food agency were observed across socio-demographic characteristics. Total food agency scores were significantly differed by education level and occupation. College/university students had the highest scores (9.99 ± 1.50), while graduates reported the lowest (9.62 ± 1.54) (*p* < 0.01). Unemployed participants showed the highest scores (9.99 ± 1.44), and employed individuals had the lowest (9.55 ± 1.54) (*p* < 0.001).

Regarding the three subscales of food agency, significant differences were most prominently observed in the structure subscale, which refers to the ability to overcome barriers. Structure scores varied significantly according to gender, education level, household composition, occupation, and household income. Men (2.92 ± 0.89) reported higher structure scores than women (2.86 ± 0.95) (*p* < 0.05). Educational attainment also significantly influenced structure scores (*p* < 0.001), with college/university graduates (2.79 ± 0.90) and those holding above bachelor’s degrees (2.75 ± 0.88) showing lower scores compared to high school graduates (3.06 ± 0.90) and college/university students (3.06 ± 0.96). Additionally, participants living in single-person households (2.76 ± 0.93) exhibited significantly lower structure scores than those living with others (3.03 ± 0.89) (*p* < 0.001). A similar pattern was evident in occupational status, where unemployed individuals had the highest structure scores (3.24 ± 0.91), followed by students (3.07 ± 0.94) and employed participants (2.69 ± 0.86) (*p* < 0.001). Household income levels further differentiated structure scores (*p* < 0.05), with participants in the lowest income group (<899 USD) reporting the highest structure scores (3.03 ± 0.91), while those in the low-income group (≥899 and <2697 USD) demonstrated the lowest scores (2.79 ± 0.90).

In contrast, self-efficacy and attitude showed statistically significant differences only in relation to household composition. Participants in single-person households reported significantly higher self-efficacy (3.51 ± 0.69) and attitude (3.43 ± 0.62) scores compared to those not living alone (*p* < 0.05) (*p* < 0.01).

### 4.2. Intention to Improve Cooking Skills and Its Underlying Motivators

For the intention to improve cooking skills, significant differences were primarily influenced by gender and household composition. Women (3.98 ± 0.94) reported a significantly higher intention to improve their cooking skills than men (3.84 ± 0.96) (*p* < 0.05). Furthermore, participants residing in single-person households (3.97 ± 0.97) exhibited significantly higher intentions compared to those not living alone (3.85 ± 0.93) (*p* < 0.05) (Table 3).

Furthermore, among the total sample of 1102 respondents, 835 reported having at least one specific motivator for the intention to improve their cooking skills. Considering multiple responses, a total of 901 unique responses were provided by these respondents to explain their motivators for improving cooking skills.

Table 4 outlines the qualitative categories of motivators underlying participants’ intentions to improve cooking skills, derived from open-ended responses and illustrated with representative quotes. The most frequently reported motivators were being influenced by media (25.3%) and gaining independence (19.5%). Other identified motivators included connecting with family and friends (16.3%), personal interest (16.2%), retaining family culinary traditions and practices (8.2%), maintaining health and weight loss (6.1%), influence of educational opportunities (4.1%), and economic reasons (3.1%).

## 5. Discussion

This study examined the food agency of young Korean adults and their intention to improve cooking skills, employing the Cooking and Food Provisioning Action Scale (CAFPAS) alongside open-ended survey responses.

Our findings underscore the prominent role of structural factors in relation to food agency. In particular, those who are highly educated, employed, or living in single-person households appear to face greater challenges in maintaining cooking-related practices without supportive environments. Participants with a college or university education and those who were employed exhibited lower overall food agency scores, primarily due to the ‘structure’ subscale, which measures the ability to overcome barriers. These results are consistent with findings from the United States, where adults with higher educational attainment and full-time employment also reported lower food agency scores on the CAFPAS [31].

Interestingly, these results contrast with earlier research that has typically identified educational attainment as a positive predictor of cooking skills [1,23]. This discrepancy may reflect that the benefits of education for food agency are less evident under the demands of modern life. While education may equip individuals with food-related knowledge, the increasing salience of structural barriers—such as time scarcity, work schedules, and limited supportive environments—may be associated with greater difficulty in sustaining cooking-related practices among young adults.

Household income was also associated with these structural conditions. Overall food agency scores generally increased with rising income, except for the lowest income group, which displayed the highest scores. This exception was primarily observed in elevated ‘structure’ subscale scores, which may reflect greater perceived flexibility in managing everyday constraints among individuals in the lowest income group, who are more likely to be unemployed. Having more available time and autonomy for cooking and food provisioning may be associated with a higher perceived ability to overcome barriers.

Previous research has often examined food practices among students and low-income populations through the lens of food insecurity. As household income is a well-established correlate of food insecurity and hunger [32], our findings provide additional descriptive context for understanding differences in food practices across income levels. For instance, a prior study found that food agency scores on the CAFPAS were significantly lower among college students experiencing marginal, low, or very low food security compared to their highly food-secure peers [33]. Similarly, although measured using a different questionnaire, students experiencing very low food security exhibited significantly lower self-efficacy in cooking and food preparation compared to their food-secure counterparts [34].

The role of structural conditions was also evident when examining participants’ living arrangements. Heightened food insecurity has recently been identified as a growing concern among young single-person households in South Korea. Although single-person households in our study had overall food agency scores comparable to those living with others, their subscale patterns differed: higher self-efficacy and attitude scores indicated greater confidence in their cooking skills and more positive perceptions of cooking, whereas lower structure scores reflected greater perceived challenges in managing everyday constraints. Even when individuals report adequate skills and motivation to cook, structural barriers—such as time scarcity, lack of supportive environments, and limited financial resources—may limit opportunities to translate these competencies into practice. The economic vulnerability of many young single-person households in South Korea may be associated with a higher risk of food insecurity and more pronounced structural constraints, pointing to the potential relevance of interventions that address both economic and environmental aspects of food agency.

In addition to education, income, and household composition, gender was also examined as a demographic characteristic. Women in our study reported greater perceived structural barriers to cooking-related practices. However, no significant differences were observed in self-efficacy, attitude, or overall food agency scores. Prevailing social norms have often suggested that women are more skilled in, and more frequently expected to assume responsibility for, domestic cooking and food provision. Based on this expectation, women in our sample might have been anticipated to report higher confidence in their cooking skills and more positive attitudes toward cooking. Nevertheless, the present findings show that gender differences in these dimensions were not statistically significant.

One possible interpretation is that marked gender differences in cooking skills may not yet be evident among young adults in their 20s. Hagmann et al. [7] reported that women in their 30s in Switzerland—where the average age at first childbirth is 31—demonstrated higher levels of cooking proficiency than women in their 20s, with these differences discussed in relation to skill acquisition following role transitions such as becoming a parent. Similarly, in South Korea, the average age of marriage for women is 31 [35] and the average age at first childbirth is approximately 33 [36]. As participants in the present study were in their 20s, only a small proportion are likely to have experienced such life transitions, which may help contextualize the limited gender differences observed in cooking-related practices.

Our findings indicate that women and individuals living alone were more likely to report a strong intention to improve their cooking skills. Among the various influences reported, media influence and the demands of independent living were the most frequently cited motivators, with 25.3% of participants mentioning platforms such as social media (YouTube, TikTok, Instagram), television programs, blogs, dramas, books, and comics. Young adulthood often coincides with a transition to independent living, during which cooking skills become more relevant. In our sample, half of the participants lived alone, which may be associated with both a greater perceived need for cooking skills and challenges in acquiring them. For these individuals, media may function as an important resource for self-directed learning and skill development. Media influence was frequently identified as a motivator, pointing to the increasing prominence of digital platforms in relation to culinary interests and learning pathways [23,24,25,37,38,39]. While media-based resources can provide accessible opportunities for skill development [23,24,26], they may also be accompanied by concerns regarding the nutritional quality of promoted content, suggesting the value of guidance and critical engagement [8,28].

Taken together, the findings of this study highlight the relevance of media-based educational approaches as a potential avenue for supporting food agency among young adults, particularly when such approaches attend to structural barriers to cooking-related practices. Previous research has consistently shown that cooking interventions can be associated with improvements in cooking self-efficacy and more positive attitudes toward cooking. For example, Rees et al. [40] reported that participation in a seven-week cooking program in Australia was associated with increased confidence and enjoyment in cooking, alongside greater engagement in domestic cooking. Similarly, Hartmann et al. [7] observed that individuals—particularly men in Switzerland—who reported greater enjoyment of cooking tended to demonstrate stronger cooking skills.

Media platforms may offer a scalable and accessible context for delivering cooking-related interventions that attend to common structural constraints. For example, Nour et al. [41] reported that tutorial cooking videos for young Australian adults, which incorporated strategies to address time and cost limitations, were associated with increased motivation to cook, particularly among individuals with initially low interest. This evidence is relevant to the present study, as time pressure, competing responsibilities, and limited resources were frequently identified as barriers to sustaining cooking practices. In this context, the widespread use of digital and social media suggests that online platforms may provide opportunities to extend the reach of cooking-related support, particularly when content is designed to be credible, engaging, and aligned with evidence-based nutritional guidance through collaboration among public health authorities, educators, and content creators.

Beyond skill acquisition, prior studies have linked stronger cooking skills with dietary quality and broader aspects of well-being. Hartmann et al. [7] reported that Swiss adults with higher cooking skills consumed more vegetables and fewer convenience foods, while Lins et al. [42] found that Brazilian adults with stronger cooking skills demonstrated greater eating competence, as conceptualized by Lohse et al. [43]. Evidence also suggests that intervention design may shape longer-term outcomes, with hands-on practice, clear instruction, and experiential components contributing to sustained engagement [44,45]. Such comprehensive approaches not only enhance cooking skills but also foster social connections, reduce food neophobia, and encourage exploration and enjoyment of a wider range of foods.

Building on this literature, the present study contributes to the field by identifying population groups—women, individuals living alone, and those who were highly educated, employed, or in the low-income group—that may experience greater structural challenges in maintaining cooking-related practices. This contribution extends existing research by emphasizing the importance of the ‘structure’ dimension of food agency, alongside self-efficacy and attitudes. Taken together, these findings suggest that efforts to support food agency in young adulthood may benefit from approaches that consider not only cooking skills and motivation, but also the everyday contexts in which cooking practices are embedded.

This study has several limitations that should be considered when interpreting the findings. First, the cross-sectional design allows only for the identification of observed trends and associations, rather than establishing temporal or causal relationships. Second, all data were self-reported, which may be subject to recall bias and social desirability bias. Third, the sample consisted solely of Korean young adults, which may limit the generalizability of the findings to other age groups or cultural contexts. In addition, although the study sample captured major demographic characteristics of Korean young adults in terms of age range and gender distribution, it was drawn from a nonprobability online panel. As a result, the findings may not be fully generalizable even to the broader population of young adults in South Korea.

Additionally, while open-ended responses provided valuable qualitative insights into participants’ motivators, the absence of in-depth qualitative interviews may have limited a more comprehensive understanding of their underlying experiences and perspectives. Finally, although the structure subscale of CAFPAS demonstrated acceptable internal consistency, the removal of items related to meal planning and family responsibilities may have reduced its conceptual breadth. As a result, the structure dimension in this study may have primarily captured immediate time-related constraints, while underrepresenting longer-term planning demands and caregiving-related responsibilities. In our sample of Korean young adults in their 20s, a large proportion of participants were unmarried and not living with family members, which may have limited the variability and salience of family-related time constraints. This limitation should be considered when interpreting findings related to structural barriers to cooking-related practices.

Future research should examine the long-term effects of media-based cooking interventions on sustained behavior change, explore strategies for tailoring content to different demographic and socioeconomic groups, and investigate how critical media literacy can be incorporated to help young adults navigate and evaluate the quality of cooking-related information encountered online. Collectively, these efforts may contribute to the development of more equitable, scalable, and sustainable approaches to strengthening food agency and improving population dietary health.

## 6. Conclusions

This study applied the concept of food agency to examine cooking-related practices and intentions to improve cooking skills among Korean young adults. The findings indicate that demographic differences in food agency were most pronounced in the structural dimension, reflecting variations in how young adults navigate time constraints and everyday contextual demands, whereas differences in cooking-related confidence and attitudes were comparatively limited. In addition, media influence and the demands of independent living emerged as salient contextual factors shaping their intentions to improve cooking skills, underscoring the importance of situating cooking-related behaviors within broader social and environmental contexts.

Taken together, these results highlight the value of moving beyond skill-based interpretations of cooking practices to consider food agency as a multidimensional construct shaped by everyday conditions. From an applied perspective, efforts to support cooking-related practices among young adults may benefit from approaches that address not only skill development but also the structural realities that constrain or enable food-related action.

By integrating food agency and motivational perspectives, this study contributes to a more nuanced understanding of cooking-related practices in young adulthood. Future research should examine how food agency and related motivations evolve across life stages and explore context-sensitive strategies to support diverse young adult populations.

## Figures and Tables

**Table 1:** General characteristics of respondents.

Classification		n (%)
Gender	Men	523 (47.5)
	Women	579 (52.5)
Education	High school graduation	144 (13.1)
	College/University student	282 (25.6)
	College/University graduation	574 (52.1)
	Above bachelor’s degree	102 (9.3)
Single-person household	Yes	552 (50.1)
	No	550 (49.9)
Occupation	Employed	592 (53.7)
	Students	313 (28.4)
	Unemployed	197 (17.9)
Monthly household income (USD) ^(1)^	<899 (Lowest)	114 (10.3)
	≥899 and <2697 (Low)	465 (42.2)
	≥2697 and <4495 (Middle)	266 (24.1)
	≥4495 (High)	257 (23.3)
Total		1102 (100.0)

^(1)^ Exchange rates: 1 USD = KRW 1112.31 as of February 2021 (Source: Economic Statistics System. https://ecos.bok.or.kr/#/ (accessed on 22 May 2024)).

**Table 2:** Differences in food agency according to participants’ socio-demographic characteristics (*n* = 1102).

Classification		Food Agency (Cooking-Related Practices) ^(2)^			
		Self-Efficacy	Attitude	Structure	Total
		Mean ± S.D.			
Gender ^(3)^	Men	3.41 ± 0.65	3.37 ± 0.58	2.92 ± 0.89	9.70 ± 1.48
	Women	3.48 ± 0.66	3.42 ± 0.59	2.86 ± 0.95	9.77 ± 1.57
	*t*-value	−1.929	−1.497	1.084 *	−0.749
Education ^(4)^	High school graduation	3.37 ± 0.65	3.34 ± 0.54	3.06 ± 0.90 ^b(5)^	9.78 ± 1.53 ^ab^
	College/University student	3.48 ± 0.63	3.46 ± 0.58	3.06 ± 0.96 ^b^	9.99 ± 1.50 ^b^
	College/University graduation	3.45 ± 0.67	3.38 ± 0.60	2.79 ± 0.90 ^a^	9.62 ± 1.54 ^a^
	Above bachelor’s degree	3.47 ± 0.67	3.44 ± 0.57	2.75 ± 0.88 ^a^	9.67 ± 1.44 ^ab^
	F-value	0.863	1.805	7.807 ***	3.829 **
Single-person Household ^(3)^	Yes	3.51 ± 0.69	3.43 ± 0.62	2.76 ± 0.93	9.70 ± 1.60
	No	3.39 ± 0.61	3.37 ± 0.55	3.03 ± 0.89	9.78 ± 1.45
	*t*-value	3.196 *	1.549 **	−4.829 ***	−0.925
Occupation ^(4)^	Employed	3.46 ± 0.67	3.39 ± 0.61	2.69 ± 0.86 ^a^	9.55 ± 1.54 ^a^
	Students	3.46 ± 0.63	3.42 ± 0.58	3.07 ± 0.94 ^b^	9.95 ± 1.51 ^b^
	Unemployed	3.38 ± 0.63	3.37 ± 0.53	3.24 ± 0.91 ^c^	9.99 ± 1.44 ^b^
	F-value	1.192	0.617	36.200 ***	10.448 ***
Monthly household income (USD) ^(1)(4)^	<899 (Lowest)	3.48 ± 0.63	3.41 ± 0.58	3.03 ± 0.91 ^b^	9.92 ± 1.49 ^b^
	≥899 and <2697 (Low)	3.42 ± 0.67	3.38 ± 0.58	2.79 ± 0.90 ^a^	9.59 ± 1.50 ^a^
	≥2697 and <4495 (Middle)	3.44 ± 0.64	3.39 ± 0.57	2.94 ± 0.93 ^ab^	9.77 ± 1.61 ^ab^
	≥4495 (High)	3.50 ± 0.65	3.43 ± 0.61	2.97 ± 0.94 ^ab^	9.89 ± 1.47 ^ab^
	F-value	0.892	0.319	3.501 *	2.818 *
Total		3.45 ± 0.65	3.40 ± 0.59	2.89 ± 0.92	9.74 ± 1.53

^(1)^ Exchange rates: 1 USD = KRW 1112.31 as of February 2021 (Source: Economic Statistics System. https://ecos.bok.or.kr/#/ (accessed on 22 May 2024)). ^(2)^ 5-point Likert scale anchored by 1: strongly disagree and 5: strongly agree. ^(3)^ *p*-value by two independent samples *t*-test. ^(4)^ *p*-value by one-way ANOVA. ^(5) a~c^: Different letters within the same column are significantly different by Duncan’s multiple range test. * *p* < 0.05, ** *p* < 0.01, *** *p* < 0.001.

**Table 3:** Differences in participants’ intention to improve cooking skills (*n* = 1102).

Classification		Intention to Improve Cooking Skills
		Mean ± S.D.
Gender ^(2)^	Men	3.84 ± 0.96
	Women	3.98 ± 0.94
	*t*-value	−2.540 *
Education ^(3)^	High school graduation	3.78 ± 1.05
	College/University student	3.91 ± 0.97
	College/University graduation	3.93 ± 0.93
	Above bachelor’s degree	3.97 ± 0.86
	F-value	1.091
Single-person Household ^(2)^	Yes	3.97 ± 0.97
	No	3.85 ± 0.93
	*t*-value	2.069 *
Occupation ^(3)^	Employed	3.97 ± 0.93
	Students	3.88 ± 0.96
	Unemployed	3.79 ± 0.99
	F-value	2.671
Monthly household income (USD) ^(1)(3)^	<899 (Lowest)	3.78 ± 1.01
	≥899 and < 2697 (Low)	3.92 ± 0.96
	≥2697 and <4495 (Middle)	4.00 ± 0.87
	≥4495 (High)	3.85 ± 0.99
	F-value	1.924
Total		

^(1)^ Exchange rates: 1 USD = KRW 1112.31 as of February 2021 (Source: Economic Statistics System. https://ecos.bok.or.kr/#/ (accessed on 22 May 2024)). ^(2)^ *p*-value by two independent samples *t*-test. ^(3)^ *p*-value by one-way ANOVA. * *p* < 0.05.

**Table 4:** Motivators behind intention to improve cooking skills: Qualitative categories (*n* = 835) ^(1)^.

Categories	n	%	Quotes
Influenced by media	228	25.3%	“Because it has become easier to access a wide range of cooking methods and recipes through TV and social media videos, I started trying to cook, and the act of cooking itself gradually became enjoyable.” “I have tried cooking after watching YouTube videos that introduce easy and tasty dishes you can make on your own.” “Watching chefs on TV made cooking look cool, and that’s how I first became interested in it.” “As mukbang and cooking shows became popular, I started to develop an interest in cooking.”
Coping with the demands of independent living	176	19.5%	“I became interested in cooking after I started living on my own during college.” “When I began living alone, I had to take care of my meals by myself. I got tired of delivery food, and when I started craving home-cooked meals, I felt like I wanted to cook and that I needed to learn how.” “After I started living on my own, I found myself wanting to cook meals that were a little tastier and fresher, even under the same conditions.”
Connecting to family and friends	147	16.3%	“I want to cook food myself and serve it to the people I love—my family or my partner.” “I feel happy when someone enjoys the food I made and genuinely tells me that it tastes good.” “I find it rewarding to cook together and share a meal with others.”
Having personal interests	146	16.2%	“I took up cooking as a hobby.” “I started wanting to make and eat delicious food by myself.” “I wanted to cook the foods I was craving, exactly to my own taste.”
Retaining family culinary traditions and practices	74	8.2%	“My mother cooked a wide variety of dishes for me from a young age, and through that, I naturally became interested in cooking.” “Everyone in my family was good at cooking, so I grew up with a strong interest in it.” “From a young age, my parents cooked meals for me themselves, and I found them tastier than food bought outside.”
Maintaining health and weight loss	55	6.1%	“When I realized that my health was getting worse as I relied mostly on delivery food and instant meals.” “My interest in healthy meals increased as I started dieting to lose weight, which led me to cook for myself.” “Because of health-related issues such as weight gain, increased fatigue, and dizziness, I became interested in cooking.”
Influenced by educational opportunities	47	4.1%	“I happened to take a one-day baking class, and I found it fun, which made me want to learn more.” “I became interested in cooking through hands-on cooking activities during my childhood.” “In middle school home economics class, we had a performance assignment that involved cooking at home. While doing that, I developed both interest and enjoyment in cooking.”
Having economic reasons	28	3.1%	“I ordered some tasty delivery food, but it was too expensive. That made me think I wanted to cook for myself, and that’s how I became interested in cooking.” “There were many foods I wanted to eat, but they were expensive, so I thought cooking would be more affordable.” “As a student, eating out or ordering food was a financial burden.”
Total	901	100	

^(1)^ Multiple responses were allowed.

## Data Availability

The anonymous dataset is available from the authors, upon reasonable request.

## Appendix A

**Table A1:** Reliability of multi-item measurements (*n* = 1102).

Variables			Mean (SD)	Cronbach’s α
Food agency	Self -efficacy	I know where to find the ingredients I need to prepare a meal	3.90 (0.79)	0.913
		I know how to use the kitchen equipment I have	3.87 (0.79)	
		I feel limited by my lack of cooking knowledge ^↑^	2.65 (0.99)	
		I am confident creating meals from the ingredients I have on hand	3.65 (0.94)	
		When I shop for food, I know how I will use the ingredients I am purchasing	3.64 (0.90)	
		I can always manage to decide what I would like to eat at any given time.	3.62 (0.89)	
		When presented with two similar products to purchase, I feel confident choosing between them	3.45 (0.89)	
		In preparing food, I can solve most problems with enough effort	3.42 (0.89)	
		Before I start cooking, I usually have a mental plan of all the steps I will need to complete	3.36 (1.00)	
		I am comfortable preparing food.	3.30 (1.04)	
		When preparing food, I am confident that I can deal with unexpected results	3.30 (0.94)	
		I am involved in daily meal preparation	3.24 (1.11)	
		When preparing food it is easy for me to accomplish my desired results	3.12 (0.94)	
		Sub total	3.48 (0.65)	
	Attitude	I think a lot about what I will cook or eat	3.76 (0.81)	0.781
		I find cooking a very fulfilling activity	3.75 (0.91)	
		I feel like cooking is a waste of effort ^↑^	3.72 (0.96)	
		I am inspired to cook for other people, like my family or friends.	3.38 (0.99)	
		Compared to other activities, cooking brings me little enjoyment ^↑^	3.38 (0.97)	
		If I try making a new type of food and it does not come out right, I usually do not try to make it again ^↑^	3.36 (1.00)	
		I feel burdened by having to cook for other people, like my family or friends ^↑^	3.23 (1.03)	
		If everything else is equal, I choose to cook rather than have food prepared by someone else	3.12 (1.06)	
		For me, cooking is just something to get through as quickly as possible ^↑^	2.88 (0.97)	
		Sub total	3.31 (0.45)	
	Structure	My job responsibilities prevent me from having the time to prepare meals ^↑^	2.93 (1.15)	0.803
		My social responsibilities prevent me from having the time to prepare meals ^↑^	2.91 (1.13)	
		I have a hard time finding enough time to prepare the food I’d like to eat ^↑^	2.84 (0.97)	
		Sub total	2.89 (0.92)	
		Total	9.68 (2.02)	

^↑^ Reverse-coded.
